# A Budget in Hand is Worth Two in the Bush

**DOI:** 10.1100/tsw.2000.20

**Published:** 2000-12-22

## Abstract

Nature leads off this week with by speculating on the possible science policy consequences of the soon-to-be-inaugurated Bush administration. The top Science story this week is the announced budget increase for the National Institutes of Health (NIH).

Now that George W. Bush has "officially" won the race for President of the United States, *Nature* chose to lead off with an article about the future of U.S. science funding. The bottom line, *Nature* concludes, is that federal support for basic research is likely to remain strong. Apparently, one of the few things that Democrats and Republicans can agree on is that basic science is good. "When there's a close balance of power, Republicans and Democrats tend to push those things they can agree upon, and that looks promising for science," said the executive director of the National Council for Science and the Environment, Peter Sundry, in the *Nature* story. *Science* agrees, at the end of their lead story on a separate topic (see below), that bipartisanship bodes well for science funding.

The *Nature* lead article also reports that Bush will soon select leaders for each of the U.S. science agencies. Likely winners? Rita Colwell should continue her six-year-long appointment as director of the National Science Foundation that began in 1999 and NASA administrator Daniel S. Goldin, appointed in 1992 by Bush the senior, also wants to keep his job, reports *Nature*. In less certain races, *Nature* speculates that Marye Anne Fox, a chemist and chancellor of North Carolina State University, is the top candidate for head the White House Office of Science and Technology Policy. And *Nature* pegs Gail Wilensky, who advised former President Bush on health issues and now works at the humanitarian organization Project HOPE, as the front-runner in the race for the powerful position of Secretary of Health and Human Services. (The secretary is the boss of the boss of the NIH.) *Science* did not speculate on likely choices for these posts.

*Science* placed the NIH budget boost in first position. Last week, the U.S. Congress approved a 14.2 percent increase for the National Institutes of Health (NIH), reports *Science*. That brings the agency budget to a record $20.3 billion, keeping the agency on track to double its budget by 2003. (Since what starting date? The article didn't say.) The "Merlot agreement," named after a bottle of wine prematurely opened to celebrate the deal last October, almost didn't happen. House Majority Leader Tom DeLay (R.-TX) led a last minute conservative attack on the bill in an attempt to "significantly reduce" the appropriation. Democrats and scientists, with wine on their lips and fearing egg on their faces, fought back. *Science* quotes Sen. Tom Harkin (D.-IA) as saying that a freeze "would be a terrible mistake that would cut [funds for] 4500 research projects." Although the Republicans "delayed" final approval for almost six weeks, Democrats hung on to their bit of the grape. Negotiators finally agreed to maintain the NIH's increase at "near-Merlot levels", reports *Science*. The conservatives won one small consolation for their efforts to stop the third consecutive double-digit increase in the NIH budget: $1 billion will be trimmed from previously-approved 2001 domestic and defense expenditures, including $9 million from the National Science Foundation. *Nature* did not cover this story.
